# The Benefit of Large Neutral Amino Acid Supplementation to a Liberalized Phenylalanine-Restricted Diet in Adult Phenylketonuria Patients: Evidence from Adult *Pah-Enu2* Mice

**DOI:** 10.3390/nu11092252

**Published:** 2019-09-19

**Authors:** Danique van Vliet, Els van der Goot, Wiggert G. van Ginkel, Martijn H. J. R. van Faassen, Pim de Blaauw, Ido P. Kema, Aurora Martinez, M. Rebecca Heiner-Fokkema, Eddy A. van der Zee, Francjan J. van Spronsen

**Affiliations:** 1 Beatrix Children’s Hospital, University Medical Center Groningen, University of Groningen, 9700 RB Groningen, The Netherlands; d01.vliet@umcg.nl (D.v.V.); w.g.van.ginkel@umcg.nl (W.G.v.G.); 2 Department of Molecular Neurobiology, Groningen Institute for Evolutionary Life Sciences (GELIFES), University of Groningen, 9700 RB Groningen, The Netherlands; e.van.der.goot@rug.nl (E.v.d.G.); e.a.van.der.zee@rug.nl (E.A.v.d.Z.); 3 Department of Laboratory Medicine, University Medical Center Groningen, University of Groningen, 9700 RB Groningen, The Netherlands; h.j.r.van.faassen@umcg.nl (M.H.J.R.v.F.); p.de.blaauw@umcg.nl (P.d.B.); i.p.kema@umcg.nl (I.P.K.); m.r.heiner@umcg.nl (M.R.H.-F.); 4 Department of Biomedicine and K.G. Jebsen Centre for Neuropsychiatric Disorders, University of Bergen, 5009 Bergen, Norway; Aurora.Martinez@uib.no

**Keywords:** phenylketonuria, *Pah-enu2* mouse model, dietary treatment, monoaminergic neurotransmitters, large neutral amino acids, adult

## Abstract

Many phenylketonuria (PKU) patients cannot adhere to the severe dietary restrictions as advised by the European PKU guidelines, which can be accompanied by aggravated neuropsychological impairments that, at least in part, have been attributed to brain monoaminergic neurotransmitter deficiencies. Supplementation of large neutral amino acids (LNAA) to an unrestricted diet has previously been shown to effectively improve brain monoamines in PKU mice of various ages. To determine the additive value of LNAA supplementation to a liberalized phenylalanine-restricted diet, brain and plasma monoamine and amino acid concentrations in 10 to 16-month-old adult C57Bl/6 PKU mice on a less severe phenylalanine-restricted diet with LNAA supplementation were compared to those on a non-supplemented severe or less severe phenylalanine-restricted diet. LNAA supplementation to a less severe phenylalanine-restricted diet was found to improve both brain monoamine and phenylalanine concentrations. Compared to a severe phenylalanine-restricted diet, it was equally effective to restore brain norepinephrine and serotonin even though being less effective to reduce brain phenylalanine concentrations. These results in adult PKU mice support the idea that LNAA supplementation may enhance the effect of a less severe phenylalanine-restricted diet and suggest that cerebral outcome of PKU patients treated with a less severe phenylalanine-restricted diet may be helped by additional LNAA treatment.

## 1. Introduction

In phenylketonuria (PKU; McKusick 261600), mutations in the gene encoding for the hepatic enzyme phenylalanine hydroxylase, that normally converts phenylalanine (Phe) into tyrosine, result in toxic accumulation of Phe in blood and brain. Following this pathophysiological concept, a severe Phe-restricted diet has been the cornerstone of treatment over the last 65 years. In addition, the principle of the severe Phe-restricted diet (limiting natural protein intake and supplementing an amino acid mixture devoid of the one(s) prior to the metabolic block) has served as an example for dietary treatment in many other inborn errors of amino acid metabolism [1]. Notwithstanding these successes, however, even in early- and continuously treated PKU patients neuropsychological outcome remains suboptimal [2,3,4,5,6], with children and adults showing some different problems [6]. The two pathophysiological mechanisms considered to be the most important in this are cerebral Phe toxicity and monoaminergic neurotransmitter impairments [7]. The latter has been shown to result from a combination of high brain Phe levels inhibiting tyrosine- and tryptophan hydroxylases and insufficient brain tyrosine and tryptophan availability, the amino acid precursors of dopamine, norepinephrine, and serotonin [8,9]. Moreover, many patients cannot adhere to the severe dietary restrictions as the diet is socially very demanding. In consequence, these patients have plasma Phe concentrations that are clearly above the recommended therapeutic range and can be accompanied by several manifestations of suboptimal brain function [10,11]. Alternative treatment strategies that particularly address these problems are, therefore, being pursued, one of these being large neutral amino acid (LNAA) treatment [12].

Both LNAA supplementation and severe dietary Phe-restriction strive at optimal cerebral amino acid and neurotransmitter biochemistry to optimize neurocognitive and psychosocial outcome through nutritional intervention, but their primary biochemical treatment targets differ. Whereas the severe Phe-restricted diet primarily aims to reduce plasma Phe concentrations in order to restore brain biochemistry, LNAA supplementation primarily aims to normalize brain amino acid and neurotransmitter concentrations by outcompeting brain Phe and favouring brain non-Phe LNAA uptake through increased plasma non-Phe LNAA availability [13]. Several studies have investigated this concept of LNAA treatment both in PKU patients and mice, all using different compositions of LNAA supplementation, protocols, and outcome measures [14,15,16,17,18,19,20,21,22]. 

To systematically investigate the biochemical working mechanisms and optimal composition of LNAA treatment for PKU patients, we have taken advantage of the existence of the PKU mouse model, which allows for direct measurement of brain amino acid and neurotransmitter concentrations. Previously, we first showed that LNAA treatment can: (1) reduce brain Phe, (2) increase brain non-Phe LNAA, and -most effectively- (3) increase brain neurotransmitter concentrations [23]. Secondly, we demonstrated that each of these brain biochemical treatment effects requires another optimal composition of LNAA supplementation [8]. Thirdly, we showed that LNAA treatment in adult PKU mice on its own is equally effective as a severe Phe-restricted diet to restore brain neurotransmitter concentrations, even though brain Phe concentrations remain significantly higher [9]. In all these studies, LNAA were supplemented to an unrestricted diet. One of the remaining questions is, therefore, whether and how LNAA treatment can be used to improve outcomes in PKU patients who cannot get their Phe concentrations within the recommended target range. In response to this clinical question and building on our previous studies on LNAA treatment in PKU mice, the present study aimed to determine the additive value of LNAA supplementation to a less severe Phe-restricted diet on brain neurotransmitter as well as brain and plasma amino acid concentrations in comparison to a severe Phe-restricted diet in adult PKU mice.

## 2. Material and Methods

### 2.1. Animals

C57Bl/6 *Pah-enu2* mice had originally been kindly provided by Prof. B. Thöny from the division of Clinical Chemistry and Biochemistry, University Children’s Hospital, Zurich, Switzerland to establish our own breeding line. From heterozygous (HTZ, +/−) mating, we obtained wild-type (WT, +/+), HTZ, and PKU (−/−) mice of both sexes. After weaning at four weeks of age, genetic characterization was performed by quantitative polymerase chain reaction (PCR) analysis on DNA extracted from ear tissue and animals were housed individually at 21 ± 1 °C on a 12-h light–dark cycle. Water and AM-II food pellets (Arie Block BV, Woerden, The Netherlands) were offered ad libitum. Animals were individually housed from the start of the experiment. In total, we used 10 WT (5 male, 5 female) and 30 PKU (15 male, 15 female). Median age at inclusion was 56 weeks (range 46–70 weeks). All procedures and treatments were carried out in strict accordance with the recommendations mentioned in the Guide for the Care and Use of Laboratory Animals of the National Institutes of Health. The Institutional Animal Care and Use Committee of the University of Groningen approved the experimental protocol before initiation of the study.

### 2.2. Experimental Design

Animals were included in the experiment at age 10–16 months. PKU mice born from different mothers were assigned to one of three different dietary treatment groups, balanced for sex and age. Body weight and food intake were measured daily during the first week of the experiment and weekly during the 5 weeks thereafter. After 6 weeks of dietary treatment, animals were euthanized by combined heart puncture and decapitation under inhalation-anesthetics with isoflurane. 

### 2.3. Experimental Diets

**Figure 1:**
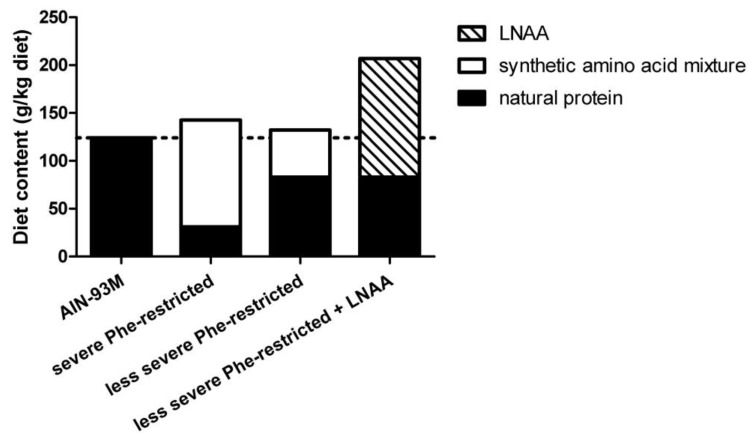
Protein contents of the various experimental diets. The dashed line indicates the total protein content of the AIN-93M control diet.

The protein contents of the experimental diets are presented in Figure 1. The basal diet was AIN-93M [24], which was administered in unadjusted form to the WT control group. The severe and less severe Phe-restricted diets were produced by reducing the amount of casein by 75% and 33% compared to the basal diet, respectively. This was compensated for by a synthetic amino acid mixture devoid of Phe, plus 20% extra for the assumed protein conversion factor [25], at the expense of cornstarch. The less severe Phe-restricted diet with LNAA was produced by adding LNAA to the 33% Phe-restricted diet at the expense of cornstarch on a weight-for-weight basis. The total amount of added LNAA in the LNAA supplemented diet was equal to the amount of protein in the basal AIN-93M diet (124.12 g/kg diet), consisting of different amounts of the various LNAA. This worked out towards a composition of supplemented LNAA consisting of: l-tyrosine (28%), l-tryptophan (17%), l-valine (14%), l-isoleucine (14%), l-leucine (14%), l-methionine (6%), l-histidine (3%), and l-threonine (3%), as previously described and used [9]. If compared to a mean natural protein intake in humans of 1 g/kg body weight/d, this corresponds to approximately 280, 170, 140, 140, 140, 60, 30, and 30 mg/kg body weight/d, respectively, in humans. Diets were prepared by Research Diet Services B.V. (Wijk bij Duurstede, The Netherlands). Results of amino acid analyses in the different diets are presented in Supplemental Table S1.

### 2.4. Biochemical Analyses

To obtain brain material for biochemical analyses, whole brains were removed and divided into cerebrum and cerebellum. The collected brain samples were all individually snap frozen in liquid nitrogen and stored at –80 °C until further preparation. Frozen cerebrum was crushed in liquid nitrogen and divided into aliquots. Frozen brain powder for amino acid measurements was processed to 20% (weight: volume (w:v)) homogenates in phosphate-buffered saline (pH 7.4), and for tryptophan, indole and catecholamine measurements to 2% (w:v) homogenates in acetic acid (0.08 M). Brain homogenates were sonified on ice at 11–12 W, centrifuged at 800 rcf for 10 min (4 °C), and the supernatant/internatant was placed on ice until further analysis. Samples were vortexed and centrifuged at 20.800 rcf for 4 min. For brain and plasma amino acid (except for tryptophan) measurements, norleucine in sulfosalicylic acid was added as an internal standard to the 20% brain homogenate (1:1, v:v) or to 50 μL plasma. Amino acid concentrations were measured with a method based on ion exchange chromatography with post-column derivatization with ninhydrin on a Biochrom 30+ analyser (Pharmacia Biotech, Cambridge, UK). 

For tryptophan and monoaminergic neurotransmitter measurements, an anti-oxidative solution was prepared in demineralised water (0.4 g/L ascorbic acid and 1.616 g/L ethylenediaminetetraacetic acid). For tryptophan and indole measurements, 25 μL of the anti-oxidative solution was added to 25 μL of the 2% brain homogenate. For catecholamine measurements, 40 μL of the anti-oxidative solution was added to 10 μL of the 2% brain homogenate. Plasma tryptophan measurements were performed using 25 μL plasma instead of 2% brain homogenate. Analysis of tryptophan and monoaminergic neurotransmitter concentrations was performed using liquid chromatography in combination with isotope dilution mass spectrometry, essentially as described by Van de Merbel et al. [26]. Monoamines and associated metabolites for which brain concentrations were assessed included dopamine, norepinephrine, 3-methoxytyramine, and normetanephrine in the catecholamine pathway, and serotonin and 5-hydroxyindoleacetic acid (5-HIAA) in the serotonergic pathway. 

### 2.5. Statistical Analyses

Statistical analyses were performed using the software IBM SPSS Statistics for Windows, Version 24.0 (Armonk, NY: IBM Corp.). All tests were performed two-sided at a significance level of α = 0.05. The effect of dietary treatment on body weight and food intake was analyzed on log-transformed data by two-way analysis of variance (ANOVA) and Tukey’s post hoc analysis with experimental group and sex as independent variables. Brain and plasma biochemistry were analyzed by one-way ANOVA and Tukey post hoc analyses on log-transformed data to induce normal distribution. In case of abnormally distributed data after log-transformation (assessed by the Shapiro–Wilk test) or unequal variances (assessed by Levene’s test), analyses were performed by Kruskal–Wallis tests and Mann–Whitney U post hoc analyses. 

## 3. Results

### 3.1. General Health and Dietary Intake

**Figure 2:**
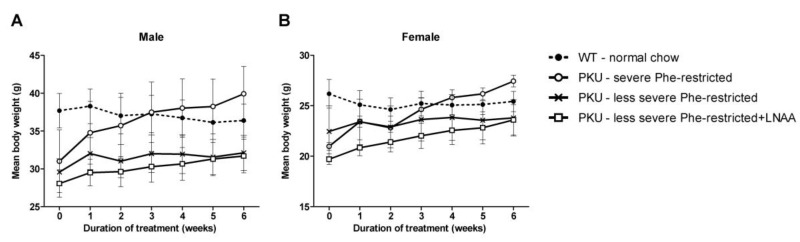
Mean body weights in male (**A**) and female (**B**) wild-type (WT) and phenylketonuria (PKU) mice on various diets during the six weeks of dietary treatment. Numbers of mice are *n* = 5 for all experimental groups. Untransformed data are expressed as mean ± standard error of the mean (SEM).

All experimental diets were well tolerated by the mice, as determined by general behaviour, body weight and food intake. The median age of the mice at the start of the experiment did not significantly differ between experimental groups (*p* = 0.76), being 55 weeks for all experimental groups (with a minimum varying from 46–55 in the different experimental groups and a maximum of 70 weeks). Figure 2 shows body weights of PKU and WT mice during the six weeks dietary treatment, separated for male and female mice. At the start of the experiment, body weight was significantly lower for female than for male mice (*p* < 0.01) and significantly lower for PKU than for WT mice (*p* < 0.01), but they did not significantly differ between PKU mice in the different experimental groups. At the end of the experiment, body weight was significantly lower in PKU mice on a less severe Phe-restricted diet with or without LNAA supplementation compared with a severe Phe-restricted diet (*p* < 0.05), but not significantly different between WT and PKU mice in each of the experimental groups. Food intake in each of the groups was highest during the first week, lowest during the second week, and relatively stable from the 3^rd^ week of treatment onwards (Supplemental Figure S1) Mean food intake during these final weeks of treatment was significantly lower in WT mice than in each of the experimental groups with PKU mice (*p* < 0.001). Also, food intake was significantly lower in female than in male mice (*p* < 0.001) without a significant interaction between sex and experimental group (*p* = 0.64).

### 3.2. Brain Monoamines 

**Figure 3:**
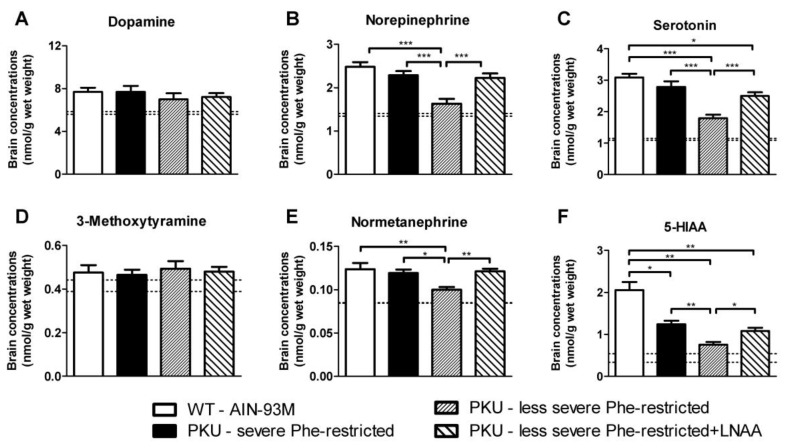
Brain concentrations of (**A**) dopamine, (**B**) norepinephrine, (**C**) serotonin, (**D**) 3-methoxytyramine, (**E**) normetanephrine, and (**F**) 5-hydroxyindoleacetic acid (5-HIAA) in WT and PKU mice after six weeks of receiving various diets. Numbers of mice are n=10 for all experimental groups. Untransformed data are expressed as mean ± SEM. * *p* < 0.05; ** *p* < 0.01; *** *p* < 0.001 (two-sided). For comparison, dashed lines represent mean brain monoamine concentrations obtained in C57Bl/6 PKU mice on AIN-93M diet in previous experiments [9,23]. Previously obtained mean brain monoamine concentrations in PKU mice on the AIN-93M diet were expressed as proportion of data in respective WT mice and then multiplied by mean brain monoamine concentrations of WT mice in the present study.

Brain monoaminergic neurotransmitter and associated metabolite concentrations in PKU mice on the various experimental diets as well as in WT mice on AIN-93M diet are shown in Figure 3. Brain dopamine and 3-methoxytyramine concentrations did not significantly differ between any of the experimental groups. Brain norepinephrine, serotonin, normetanephrine, and 5-HIAA, however, were all significantly impaired in PKU mice on the less severe Phe-restricted diet when compared to WT mice, and improved or even completely restored on both the less severe Phe-restricted diet + LNAA as well as on the severe Phe-restricted diet. The cerebral deficiencies of norepinephrine and normetanephrine in PKU mice on the less severe Phe-restricted diet, being 66% (*p* < 0.001) and 80% (*p* < 0.01) of concentrations in WT mice on the AIN-93M diet, respectively, were restored to concentrations that did no longer significantly differ from WT concentrations on both the less severe Phe-restricted diet + LNAA (*p* = 0.449 and *p* = 0.10, respectively) and the severe Phe-restricted diet (*p* = 0.690 and *p* = 0.95, respectively). In addition, the cerebral deficiencies of brain serotonin and 5-HIAA in PKU mice on the less severe Phe-restricted diet, being, respectively, 58% (*p* < 0.001) and 37% (*p* < 0.001) of concentrations in WT mice on AIN-93M diet, were improved to 81% and 52% of concentrations in WT mice on the less severe Phe-restricted diet + LNAA, and to 90% and 60% of concentrations in WT mice on the severe Phe-restricted diet.

### 3.3. Brain Large Neutral Amino Acids (LNAA) 

**Figure 4:**
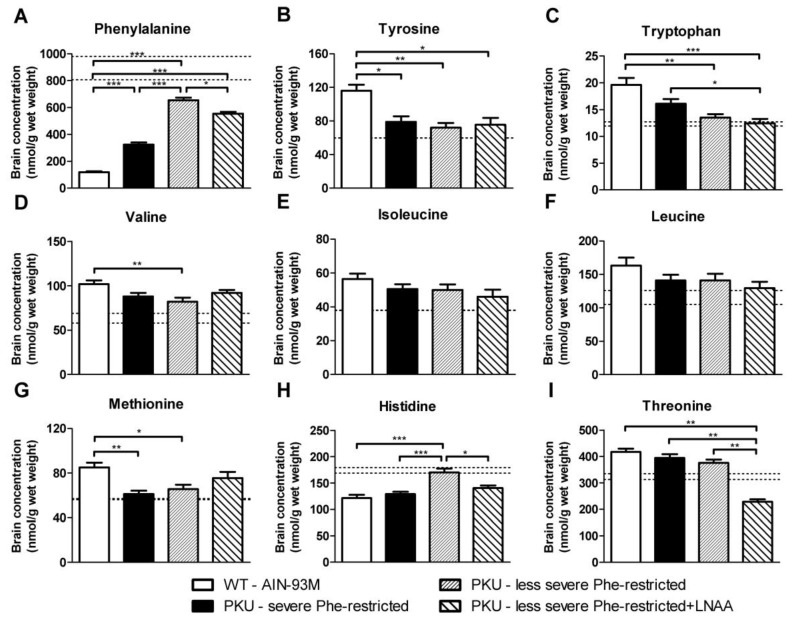
Brain concentrations of (**A**) phenylalanine, (**B**) tyrosine, (**C**) tryptophan, (**D**) valine, (**E**) isoleucine, (**F**) leucine, (**G**) methionine, (**H**) histidine, and (**I**) threonine in WT and PKU mice after six weeks of receiving various diets. Numbers of mice are *n* = 10 for all experimental groups. Untransformed data are expressed as mean ± SEM. * *p* < 0.05; ** *p* < 0.01; *** *p* < 0.001 (two-sided). For comparison, dashed lines represent mean brain amino acid concentrations obtained in C57Bl/6 PKU mice on AIN-93M diet in previous experiments [21,23]. Previously obtained mean brain amino acid concentrations in PKU mice on AIN-93M diet were expressed as proportion of data in respective WT mice and then multiplied by mean brain amino acid concentrations of WT mice in the present study.

Brain concentrations of individual LNAA in PKU mice on the various experimental diets as well as in WT mice on AIN-93M diet are shown in Figure 4. Brain Phe concentrations in PKU mice on all of the experimental diets were significantly higher than in WT mice (*p* < 0.01). When comparing the different experimental diets in PKU mice to one another, brain Phe concentrations in PKU mice on the less severe Phe-restricted diet + LNAA and on the severe Phe-restricted diet were 85% and 49%, respectively, of those in PKU mice on the less severe Phe-restricted diet without LNAA (*p* < 0.05 and *p* < 0.001).

Brain concentrations of other LNAA all showed different responses to the various experimental diets in PKU mice. Brain tyrosine and tryptophan concentrations were significantly lower in PKU mice on the various Phe-restricted diets compared with WT mice, and did not improve on LNAA supplementation. Brain methionine concentrations in PKU mice on both the less severe Phe-restricted diet and the severe Phe-restricted diet were significantly lower compared with WT mice (*p* < 0.01 and *p* < 0.05, respectively), but not when LNAA were supplemented (*p* = 0.43). In contrast, brain threonine concentrations were significantly lower in PKU mice on the less severe Phe-restricted diet + LNAA compared with both Phe-restricted diets without LNAA supplementation as well as with WT mice (*p* < 0.01), while brain histidine concentrations were significantly higher in PKU mice on a less severe Phe-restricted diet compared with all other treatment groups (*p* < 0.05).

### 3.4. Plasma LNAA 

**Figure 5:**
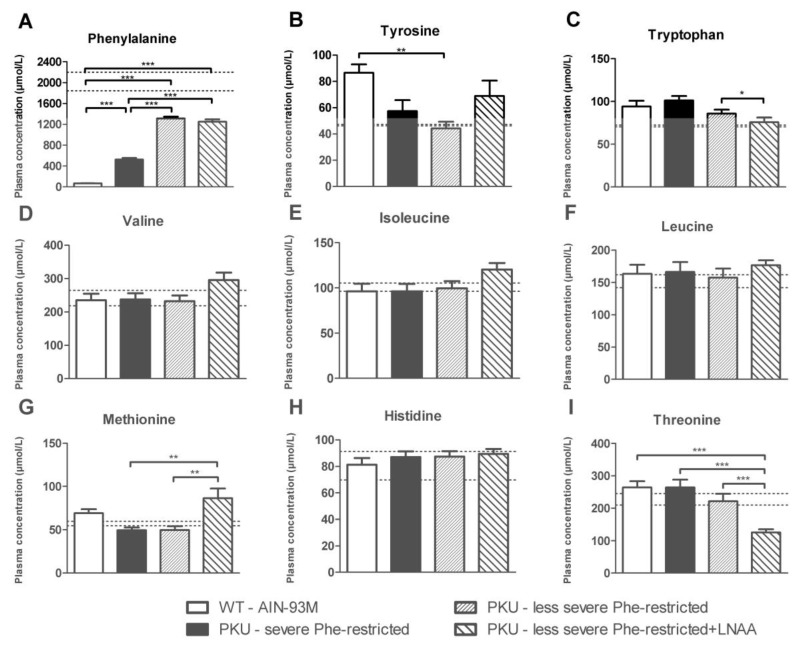
Plasma concentrations of (**A**) phenylalanine, (**B**) tyrosine, (**C**) tryptophan, (**D**) valine, (**E**) isoleucine, (**F**) leucine, (**G**) methionine, (**H**) histidine, and (**I**) threonine in WT and PKU mice after six weeks of receiving various diets. Numbers of mice are *n* = 10 for all experimental groups. Untransformed data are expressed as mean ± SEM. * *p* < 0.05; ** *p* < 0.01; *** *p* < 0.001 (two-sided). For comparison, dashed lines represent mean plasma amino acid concentrations obtained in C57Bl/6 PKU mice on the AIN-93M diet in previous experiments [21,23]. Previously obtained mean plasma amino acid concentrations in PKU mice on the AIN-93M diet were expressed as proportion of data in respective WT mice and then multiplied by mean plasma amino acid concentrations of WT mice in the present study.

Plasma concentrations of individual LNAA in PKU mice on the various experimental diets as well as in WT mice on AIN-93M diet are shown in Figure 5. Plasma Phe concentrations in PKU mice on each of the experimental diets were significantly higher than in WT mice on AIN-93M diet (*p* < 0.001). Plasma Phe concentrations were significantly lower on the severe Phe-restricted diet if compared to both less severe Phe-restricted diets (*p* < 0.001), but they did not significantly differ between the less severe Phe-restricted diet with and without LNAA supplementation. 

Plasma concentrations of other LNAA did not significantly differ between PKU mice on one of the experimental diets and WT mice on the AIN-93M diet except for plasma tyrosine concentrations that were significantly lower in PKU mice on the less severe Phe-restricted diet (*p* < 0.01) and plasma threonine concentrations that were significantly lower in PKU mice on the less severe Phe-restricted diet + LNAA compared to WT mice (*p* < 0.001). While plasma concentrations in PKU mice on the less severe Phe-restricted diet + LNAA tended to be higher compared to both Phe-restricted diets without LNAA supplementation, for some of the LNAA this was only statistically significant for methionine (*p* < 0.01). In contrast, in PKU mice on the less severe Phe-restricted diet + LNAA, plasma tryptophan concentrations were significantly lower than on the corresponding diet without LNAA supplementation (*p* < 0.05), while plasma threonine concentrations were significantly lower compared to both Phe-restricted diets without LNAA supplementation (*p* < 0.01).

## 4. Discussion

Responding to the fact that, especially adult, PKU patients often cannot adhere to the severe Phe-restricted diet well enough to ensure their plasma Phe concentrations are within the recommended target range [25], and building on our previous studies on LNAA treatment in PKU mice, the present study aimed to determine the possible benefit of LNAA supplementation to a less severe Phe-restricted diet on plasma and brain amino acid and brain neurotransmitter concentrations in comparison to a severe Phe-restricted diet in adult PKU mice. The two most important findings of this study were that, when LNAA supplementation was added to a less severe Phe-restricted diet: (1) brain norepinephrine and serotonin concentrations significantly increased and brain Phe concentrations reduced, and (2) brain norepinephrine and serotonin concentrations were equally effectively restored, but brain Phe concentrations were less effectively reduced when compared to a severe Phe-restricted diet. 

Before discussing these results in further detail, we address some methodological issues. Firstly, this study was performed in 10- to 16-month-old PKU mice as a model for (late-)adult PKU patients. Although it should be noted that these results obtained in PKU mice cannot directly be translated to the clinical setting, our previous studies showed that the PKU mouse provides a good model to investigate the brain biochemical effects of LNAA treatment in PKU [8,23]. Also, of particular advantage in the present study, such a mouse model is not subject to bias of differences in compliance between dietary treatments. Secondly, the (less) severe Phe-restricted diets used here were designed to be nutritionally adequate with the synthetic Phe-free amino acid mixture, compensating for the natural protein restriction. In clinical practice, however, if patients themselves liberalize their Phe-restricted diet, this compensation may not always be the case. Thirdly, we did not include PKU mice on an unrestricted and non-supplemented (AIN-93M) diet in this study, but used the results on brain and plasma amino acid and brain monoamine concentrations in young and adult C57Bl/6 PKU mice on such a diet from our previous studies that showed consistent results [9,23]. To enable comparison, we added those previously reported data to Figure 3, Figure 4 and Figure 5 (dashed lines).

The present study showed that substituting the synthetic Phe-free amino acid mixture for LNAA supplementation in PKU mice on a less severe Phe-restricted diet significantly increased brain norepinephrine and serotonin, and reduced brain Phe concentrations. In line with the concept of LNAA treatment, the less severe Phe-restricted diet + LNAA differed from the corresponding diet without LNAA treatment with respect to both the total amount and composition of the amino acids being supplemented [13,23]. Previous studies in PKU mice showed that the particular effectiveness of LNAA treatment to improve brain monoamine concentrations is due to its combined effects of reducing brain Phe and increasing precursor (tyrosine and tryptophan) availability [8,23]. In addition, based on their findings on peripheral monoaminergic neurotransmitter markers in response to LNAA supplementation in PKU patients, Yano et al. suggested that for serotonergic neurotransmitters the effect of brain Phe seems to be most important, while for dopaminergic neurotransmitters the effect of tyrosine availability seems to be the most important [22,27]. Following this hypothesis, the here used LNAA supplement was primarily enriched in tyrosine, tryptophan, and the branched-chain amino acids. Besides, all other non-Phe LNAA were supplemented as well, including methionine that has been associated with increased tetrahydrobiopterin (BH_4_) levels and thereby might contribute to increased brain monoaminergic neurotransmitter synthesis as well [28,29]. This LNAA supplementation regimen, when added to a less severe Phe-restricted diet in PKU mice, effectively increased brain norepinephrine and serotonin, and reduced brain Phe concentrations, while brain tyrosine and tryptophan concentrations were not increased. This discrepancy with previous findings might be explained by the fact that in previous LNAA studies that required both reduced brain Phe and increased brain tyrosine and tryptophan availability for optimal improvement of brain neurotransmitter concentrations, LNAA were added to an unrestricted instead of a less severe Phe-restricted diet, so that brain Phe concentrations were higher [8,9,23]. Interestingly, and in accordance with previous studies [8,9,23], brain concentrations of norepinephrine were significantly impaired in PKU mice and could be effectively increased on LNAA treatment, while brain dopamine concentrations remained largely unaffected. An explanation for these findings might be that phenethylamine, the monoamine derivative of Phe, is a competitive inhibitor of norepinephrine synthesis [30]. Furthermore, our results are also in accordance with findings in tyrosine hydroxylase-deficient mice in which norepinephrine is also largely impaired and at earlier age compared with dopamine, suggesting that the homeostasis mechanisms for dopamine synthesis, degradation and release are more strictly controlled than those for norepinephrine [31]. At least, the combination of findings in the different LNAA studies shows that the relationship between brain Phe, tyrosine, tryptophan and neurotransmitter concentrations in PKU is complex and warrants further investigation. While the exact relationships between plasma LNAA, brain LNAA, and brain neurotransmitters remain to be established, both brain Phe and neurotransmitters have been ascribed to be important for brain functioning in PKU [32]. Based on these associations, our results at least suggest that cerebral outcome of PKU patients treated with a less severe phenylalanine-restricted diet may be helped by additional LNAA treatment.

When it comes to treating adults with PKU, one of the main issues is the question of how the effects of treatment relate to the associated treatment burden. Both the European and US guidelines on PKU management advocate lifelong treatment, but differ with respect to the upper target plasma Phe levels for adult PKU patients as the primary therapeutic target [1,33]. Many adult PKU patients cannot adhere to the severe Phe-restricted diet and liberalize their diet, resulting in plasma Phe concentrations clearly above the target levels of both guidelines [10,11]. Comparing the LNAA supplemented less severe Phe-restricted diet with the severe Phe-restricted diet in view of their treatment effects, both treatments were similarly effective in restoring brain neurotransmitter concentrations (that can be considered to be the most important brain biochemical parameter of PKU symptoms in adults), while the severe Phe-restricted diet was most effective to reduce brain Phe concentrations (that is often considered to be the most important brain biochemical parameter to PKU symptoms in children). With regard to the associated treatment burden, the severity of natural protein restriction (usually considered the most burdensome aspect of the current dietary treatment) in the less severe Phe-restricted diet with LNAA was only about 44% of the severity of the natural protein restriction in the severe Phe-restricted diet, while the amount of amino acid supplementation (as essential amino acid mixture and LNAA supplementation) was “only” increased by 11% compared to that in the severe Phe-restricted diet. Taken together, LNAA supplementation to a less severe Phe-restricted diet thus seems to provide a favorable alternative to the severe Phe-restricted diet for those PKU patients who cannot keep their plasma Phe concentrations within the recommended target range. When comparing the results obtained with LNAA supplementation to a less severe Phe-restricted diet in the present study to those with LNAA supplementation to an unrestricted diet in previous studies [8,9], both applications of LNAA treatment seem to be similarly effective. This also accounts for the plasma Phe concentrations, as plasma Phe concentrations in PKU mice on a less severe Phe-restricted diet with LNAA were comparable to those on LNAA supplementation to an unrestricted diet in a previous study. Therefore, further studies should determine the possible additive effect of a less severe Phe-restricted diet to LNAA treatment.

Previous studies in PKU mice demonstrated that the opposing effects on Phe, tyrosine, and tryptophan concentrations in plasma and the brain are key to the success of LNAA treatment. While the present study also showed that LNAA supplementation to a less severe Phe-restricted diet could effectively improve brain monoaminergic neurotransmitters, such an effect on brain tyrosine and tryptophan was not observed. At the same time, however, data also showed that the improved brain monoaminergic neurotransmitter concentrations were not solely due to the effect on brain (nor plasma) Phe concentrations (Supplemental Figure S2). Thereby, these results urge for (1) further studies on the complex system of amino acids and monoaminergic neurotransmitters in PKU and (2) the identification of alternative biomarkers that better correlate to brain monoaminergic neurotransmitter concentrations to be used for monitoring of treatments that do not primarily work by reducing plasma Phe concentrations such as LNAA supplementation. This also accounts for the relationship between plasma LNAA and brain Phe concentrations. In the present study, brain Phe concentrations were significantly reduced on LNAA supplementation to a less severe Phe-restricted diet, while plasma Phe concentrations were unchanged. In contrast, plasma tyrosine, branched-chain amino acid, and methionine concentrations were all increased. The most interesting in this is that plasma methionine concentrations increased a lot. Even more interesting, however, is the fact that this seems to be only one factor that influences the brain LNAA levels. Whether this means that there are other mechanisms than LAT1 and B0AT1 important in the exchange of LNAA from blood to brain remains to be understood. Moreover, the effects of LNAA treatment on threonine concentrations have not been fully understood either. Threonine concentrations in both plasma and brain were significantly reduced on the LNAA-supplemented compared to the non-supplemented less severe Phe-restricted diet despite inclusion of threonine in the LNAA supplement. Previously investigated LNAA supplementation regimens with higher threonine dosage showed strongly increased threonine concentrations in both plasma and brain [8], suggesting that threonine metabolism is a highly sensitive system. While the significance of these alterations in threonine concentrations (on LNAA treatment) in PKU requires further investigation, at least in the present study, brain serine and glycine concentrations were not affected by the reduced threonine concentrations.

In view of a clinical application of LNAA treatment in PKU, the current finding in PKU mice that LNAA supplementation to a less severe Phe-restricted diet could improve brain biochemistry, supports previous data that LNAA supplementation could offer a promising treatment strategy to improve neurocognitive and psychosocial outcomes in adult PKU patients [19,20,21]. Because of the large number of, especially, adults with PKU who cannot adhere to the severe dietary restrictions resulting in plasma Phe concentrations above the recommended target range, and the importance of brain neurotransmitter impairments to their symptoms, LNAA treatment can be expected to offer a real health benefit especially for adults with PKU. Another relatively new dietary treatment modality for PKU that shares some of these principles with LNAA treatment, and that has already proven its value in clinical practice, includes glycomacropeptide (GMP) [34,35]. Similar to LNAA treatment, GMP provides an alternative to the original Phe-free amino acid mixture [36]. In contrast, where GMP exploits the benefits of a palatable and low-Phe intact protein [37], LNAA treatment does not bring the benefits of an intact protein source but is shown to be able, based on its specific composition, to improve brain biochemistry. To further increase the effectiveness and tolerability of LNAA treatment, future research should try to optimize LNAA treatment so that (1) it can further reduce brain Phe concentrations in addition to its (already optimal) effect on brain neurotransmitters, and (2) it will require a smaller total amount of LNAA to be supplemented. In relation to the latter, the effects of a possibly increased amino acid load to renal functioning, exerted by LNAA treatment, deserves further attention [38,39]. Another possible future treatment target for PKU that was only recently investigated in a preclinical setting and shares some pathophysiological concepts with LNAA treatment involves blockage of the SLC6A19 transporter which is responsible for intestinal uptake as well as renal reabsorption of amino acids, especially of LNAA. Blockage of this transporter in a PKU mouse model induced hyperaminoaciduria and selective reduction of plasma as well as brain Phe concentrations, while plasma and brain concentrations of other amino acids were relatively unaffected [40]. At least in theory, SLC6A19 blockage and LNAA treatment offers a promising treatment combination that deserves further research.

## 5. Conclusions

To conclude, the present study aimed at determining the added/protective value of LNAA supplementation to a less severe Phe-restricted diet on plasma and brain amino acid and neurotransmitter concentrations in comparison to a severe Phe-restricted diet in adult PKU mice. Thereby, it responds to the fact that many adult PKU patients cannot adhere to the severe dietary restrictions and builds on our previous studies on LNAA treatment in PKU mice. This study demonstrated that LNAA supplementation to a less severe Phe-restricted diet improved brain neurotransmitter and Phe concentrations. Compared to a severe Phe-restricted diet, it was equally effective to restore brain norepinephrine and serotonin concentrations even being less effective to reduce brain Phe concentrations. These results make LNAA supplementation a promising treatment strategy to improve neuropsychological outcomes in adults with PKU who have liberalized their Phe-restricted diet. 

This treatment approach, combining a less-aggressive Phe restriction with LNAA supplementation, might be seen as an avenue for future research. During recent years, many new drug treatment strategies have been proposed in PKU patients, including various forms of gene therapy and some are really very promising (Lichter-Koncieki and Vockley 2019). Yet, all those have their own drawbacks, either financial, not for all patients, safety concerns, need for other co-medicine etc. In addition to this, understanding the phenomenon of dietary supplementation of the correct amino acids in the optimal dose might not only help PKU but also the treatment of other patient groups with a defect in amino acid metabolism.

## Supplementary Materials

The following are available online at https://www.mdpi.com/2072-6643/11/9/2252/s1: Figure S1: Mean weekly food intake per body weight in male (A) and female (B) WT and PKU mice on various diets during the six weeks of dietary treatment, Figure S2: Brain serotonin (A,C) and norepinephrine (B,D) concentrations in relation to plasma (A,B) and brain (C,D) phenylalanine concentrations in PKU mice on various experimental diets, Table S1: Nutritional content of the experimental diets (g/kg diet or kcal/kg diet).
