# Supplementary material for: The Benefit of Large Neutral Amino Acid Supplementation to a Liberalized Phenylalanine-Restricted Diet in Adult Phenylketonuria Patients: Evidence from Adult Pah-Enu2 Mice
Source: Nutrients. 2019 Sep 19;11(9):2252. doi: 10.3390/nu11092252 (PMC6770592; doi:10.3390/nu11092252)
Supplement: Supplementary file 1 [file nutrients-11-02252-s001.pdf]

Supplemental Table S1. Nutritional content of the experimental diets (g/kg diet or kcal/kg diet)

**Nutritional content ***		**AIN-93M**	**Phe-restricted**	**semi Phe-restricted**	**semi Phe-restricted + LNAA**
**Carbohydrates**		674	668	672	606
**Fat**		41	41	41	41
**Dietary fibre**		50	50	50	50
**Protein**		124	143	132	207
**(% intact / % amino acids)**		(100/0)	(22/78)	(63/37)	(40/60)
**Energy**		3662	3615	3681	3618
**%**	Carbohydrates	73.6	73.9	73.0	67.0
	Fat	10.1	10.0	10.0	10.1
	Dietary fibre	2.7	2.8	2.7	2.8
	Protein	13.6	13.3	14.3	20.1
**Amino acids ****					
**LNAA**	L-Phenylalanine	6.6	1.8	4.5	3.9
	L-Tyrosine	6.6	9.2	7.8	37.4
	L-Valine	8.0	7.8	7.9	21.9
	L-Isoleucine	6.0	6.7	6.4	19.1
	L-Leucine	12.3	12.0	12.2	23.8
	L-Methionine	1.8	0.5	0.9	6.2
	L-Histidine	3.5	4.0	3.7	5.3
	L-Threonine	5.8	5.4	5.5	6.6
**non-LNAA**	L-Aspartic acid	10.1	9.9	9.7	5.8
	L-Serine	7.9	17.4	11.6	4.6
	L-Glutamic acid	29.3	7.7	19.5	16.8
	Glycine	2.4	7.9	4.7	1.4
	L-Alanine	4.3	5.9	4.9	2.6
	L-Lysine	11.2	10.1	10.7	6.7
	L-Arginine	4.7	9.0	6.5	2.7

Contents are not shown for L-Tryptophan, L-Proline, and L-cyst(e)ine, as these were not measured due to technical limitations. *Mineral and vitamin premixes were also included in accordance with the composition of the AIN93M diet [24]. **Dietary contents as measured in samples of prepared food pellets by first hydrolyzing the protein in the food pellets with hydrochloric acid. Then, the amount of the separate amino acids in the hydrolysate was determined by ultra fast liquid chromatography (UFLC) using a pre-column derivatization with o-phtaldialdehyde and fluorimetry as detection.

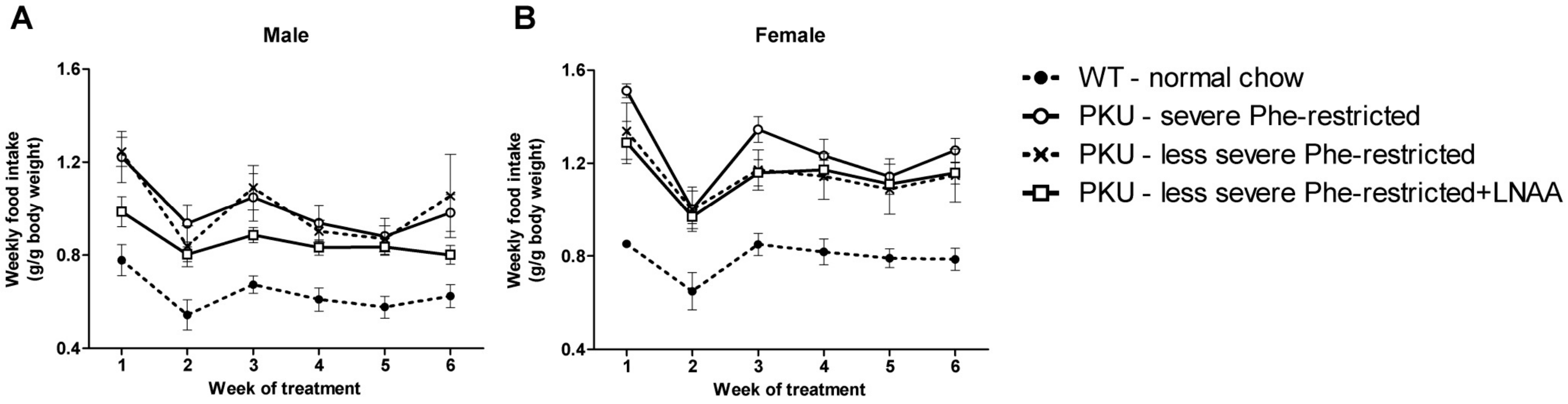

Supplemental Figure S1. Mean weekly food intake per body weight in male (A) and female (B) WT and PKU mice on various diets during the six weeks of dietary treatment. Numbers of mice are n=5 for all experimental groups. Untransformed data are expressed as mean ± SEM.

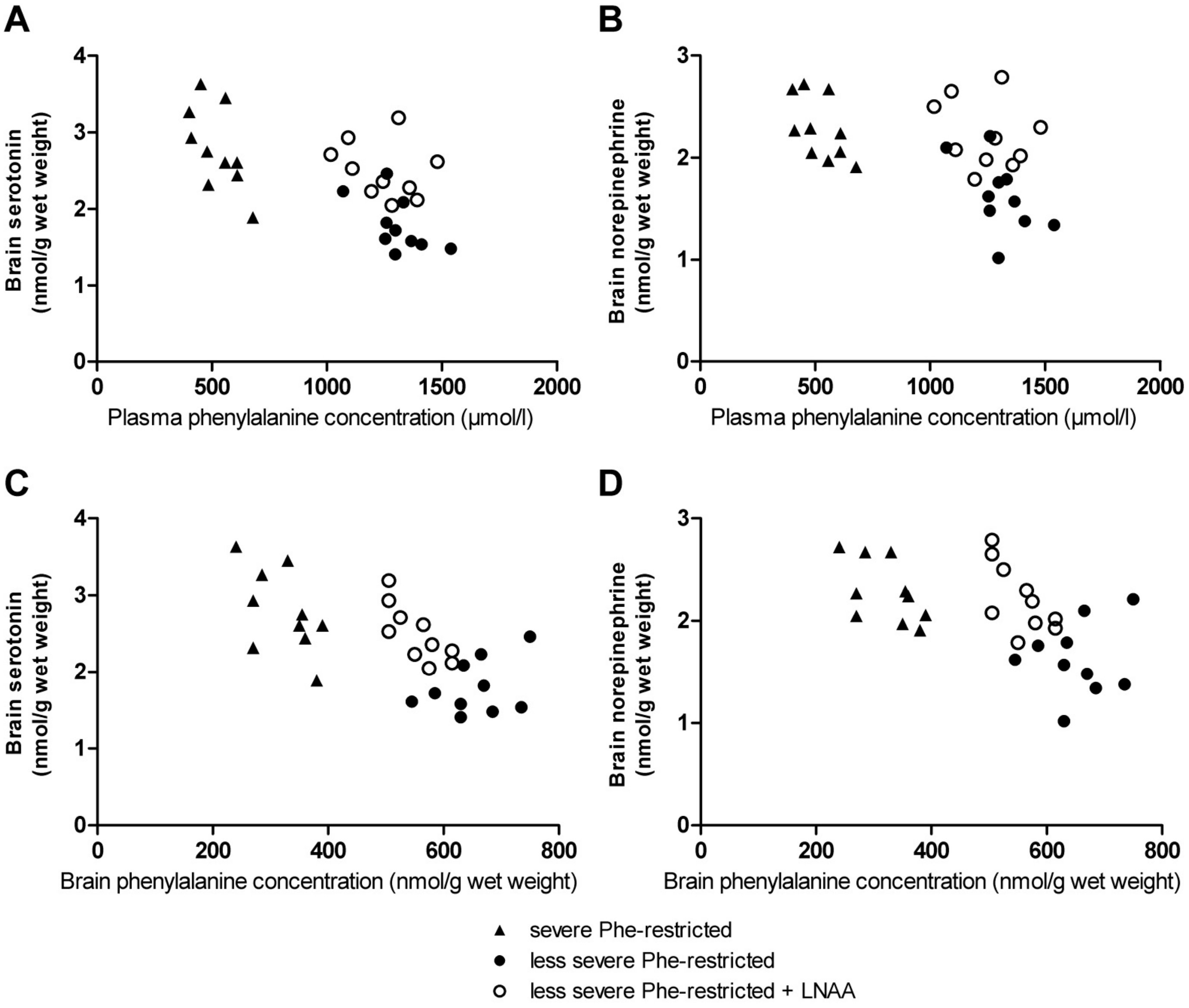

Supplemental Figure S2. Brain serotonin (A,C) and norepinephrine (B,D) concentrations in relation to plasma (A,B) and brain (C,D) phenylalanine concentrations in PKU mice on various experimental diets. Numbers of mice are n=10 for all experimental groups.
